# Multifaceted health coaching intervention for cardiovascular risk prevention – exploratory qualitative study of Chinese clients' perspectives

**DOI:** 10.1186/s12875-025-02957-0

**Published:** 2025-08-04

**Authors:** Xue Yang, Gengcong Qu, Lawrence Luk, Phoenix K. H. Mo, Benjamin H. K. Yip, Samuel Y. S. Wong, Carmen K. M. Wong

**Affiliations:** Jockey Club School of Public Health and Primary Care, Faculty of Medicine, The Chinese University of Hong Kong, Hong Kong, China

**Keywords:** Cardiovascular diseases, Prevention, Health coaching, Lifestyles, Behavioral change, Qualitative study

## Abstract

**Background:**

Cardiovascular diseases, a leading cause of death globally and in Hong Kong, lead to disability, mood changes, and increased healthcare burden. Lifestyle modifications can prevent these diseases. Health coaching, a client-centered approach, aids in behavior change. Despite its promise, cultural nuances in Hong Kong, like traditional diets and collectivist values, may influence health coaching effectiveness. Understanding these factors is crucial for tailored and effective cardiovascular disease prevention in the local Chinese population.

**Methods:**

Eighteen participants were recruited from the We WATCH project, a health coaching program for cardiovascular disease prevention in middle-aged adults (35–59 years) at post-program. Purposive sampling was used regarding gender, age and lifestyle domains. Three semi-structured focused-group interviews were conducted. Interviews followed a guide focusing on lifestyle changes and health coaching experiences. Thematic analysis, based on Braun and Clarke’s approach, identified emergent themes.

**Results:**

The study revealed challenges faced by middle-aged individuals in Hong Kong in adopting healthy habits due to various barriers. Comfort foods and inactivity hinder behavioral changes. The end of health coaching programs posed a barrier. Participants cited facilitators from health coaching for promoting healthy behaviors. Clients noted health coach characteristics impacting effectiveness. A mix of online and offline channels was preferred for health coaching modality in Hong Kong for a more versatile approach.

**Conclusions:**

Psychological, behavioral, and cultural factors impact health decisions. Tailored health coaching in Hong Kong and similar cultures should consider unique cultural and environmental contexts (e.g., family-based health coaching, exploring indoor exercises).

**Supplementary Information:**

The online version contains supplementary material available at 10.1186/s12875-025-02957-0.

## Background

Cardiovascular diseases (CVDs) are one of the most burdensome diseases with high mortality. The CVD epidemic is especially pertinent in Asia, contributing to 60% of the total 18.6 million cardiovascular deaths recorded worldwide in 2019 [1]. A recent study estimated that crude cardiovascular mortality was expected to rise 91.2% between 2025 and 2050 in Asia [2]. It has become the fourth leading cause of death in Hong Kong [3]. A cross-sectional multicenter survey in Hong Kong adults reported that only 0.6% participants had attained ideal cardiovascular health [4].

CVDs are preventable by modifying lifestyles. The American College of Lifestyle Medicine highlights six important lifestyles to improve health, including healthy eating, increasing physical activity, developing strategies to manage stress, cessation of tobacco, healthy relationships and improving sleep [5]. However, uptake or maintenance of the changes by individual efforts has been found challenging [6, 7]. A number of behavioral change theories (e.g., the stage of change theory) and growing knowledge of the barriers and facilitators (e.g., self-efficacy) of lifestyle behavior change have been identified [8–11].

Health coaching may facilitate behavioral changes by addressing these factors. Health coaching is a collaborative, client-centered, and goal-oriented partnership. In health coaching, a coach helps a person to identify personal strengths and motivates them to focus on lifestyle behaviors they want to change [12]. This is in contrast to the biomedical model in which patients are to be compliant with provider-directed and provider-controlled health care. Research into the effectiveness and feasibility of health coaching in rehabilitation and prevention has shown promise [13], but results have been inconsistent [14]. Although health coaching can be helpful for cardiovascular disease prevention, qualitative research is lacking on specific factors of uptake or maintenance of healthy lifestyles relating to health coaching prevention programs [15, 16]. Our scoping review identified six qualitative studies that were conducted in community adults after finishing health coaching programs for chronic disease prevention in South Africa, Europe, Australia, or America [17–22]. Goal setting, support, information, self-efficacy and motivation were often mentioned to be facilitators of lifestyle change. However, we did not identify any related studies conducted in Hong Kong community or Chinese culture. Community or culture related factors remain unknown.

It is crucial to understand these factors in local clients in Hong Kong. Hong Kong is a unique mix of East and West; despite the dominance of traditional Chinese values, there is an unmistakable Western influence, legacy of its time as a British colony until 1997, when the region was ceded back to China. The Chinese society is typically collectivistic, and behavior is strongly influenced by Confucian values and beliefs, which stress the importance of individuals conforming with the expectations of their role and position in society and compliance with shared norms over individual needs and desires [23, 24]. These unique experiences, beliefs, and values may shape their views on wellness and lifestyles and bring challenges for health coaching. For example, in collectivistic societies, the family unit plays a critical role in health-related decisions (e.g., food choice and dietary preference, professional help-seeking decision) and practices and individual needs are considered secondary [25]. These may shape values and motives of behavioral changes of the local clients. The dynamic nature of health coaching demands a deep understanding of the diverse populations. Recognizing the nuances of each community or culture is essential for crafting coaching programs that resonate culturally, fostering effective communication, empowering clients, and establishing trust-key elements in successful health coaching endeavors. By identifying and addressing community and culture-specific barriers, health coaches can enhance their ability to support clients from diverse backgrounds, aiding them in reaching their health objectives.

The current qualitative study aimed to fill this gap by exploring the facilitators and barriers to behavior change following real-life experiences of a hybrid-approach (face-to-face and remote) health coaching prevention program for cardiovascular diseases in Chinese population in Hong Kong. The health coaching was based on the stage of change theory [26], cognitive behavioral therapy [27] and motivation interviewing [28]. The lived experience of individuals who received health coaching and the factors that influenced their health-risk behaviors can be used to tailor coaching programs for cardiovascular disease prevention and therefore has implications for clinical practice in the local community.

## Methods

### Study design

Ethical approval of this study was obtained from the clinical ethics review committee of the corresponding author’s university. Participants were recruited from a larger project (We WATCH) conducted by our team, which was undertaken to evaluate the effectiveness of health coaching intervention in a middle-aged population (aged 35 to 59) for cardiovascular diseases prevention. The intervention inclusion criteria included adults aged 35 to 59 who were at low to moderate risk of cardiovascular diseases (Tang, et al., under review). Those who had cardiovascular disease or regular follow up/usual care (high risk) were excluded. During the project, 18 health coaches with various health professional backgrounds (e.g., dieticians, counselors, physical trainers) from four collaborating NGOs in Hong Kong took part in the 88-hour health coaching training course and individual and group supervisions. The trainers included a health psychologist (XY), a clinician (CW) and a counsellor (LL) and all of them are certified health coaches. The intervention included 6 to 12 individual sessions (depending on the risk level) of health coaching meetings in combination with the use of digital devices (website, mobile app, and health watch) over a 6-month period. Each session lasted 30-60 minutes. It was delivered by a hybrid approach including face-to-face meetings and remote sessions via Zoom, phone, or WhatsApp. The health coaching consisted of 5 steps: 1) individual health & lifestyle assessment and report, 2) establishment of individualized health goals, 3) client wellness assessment, 4) intervention guide, 5) shared decision making on smart goals. Health coaches used techniques of motivational interviewing and cognitive behavior therapy to motivate the participants for lifestyle change and maintenance.

#### Interview procedure

**Fig. 1:**
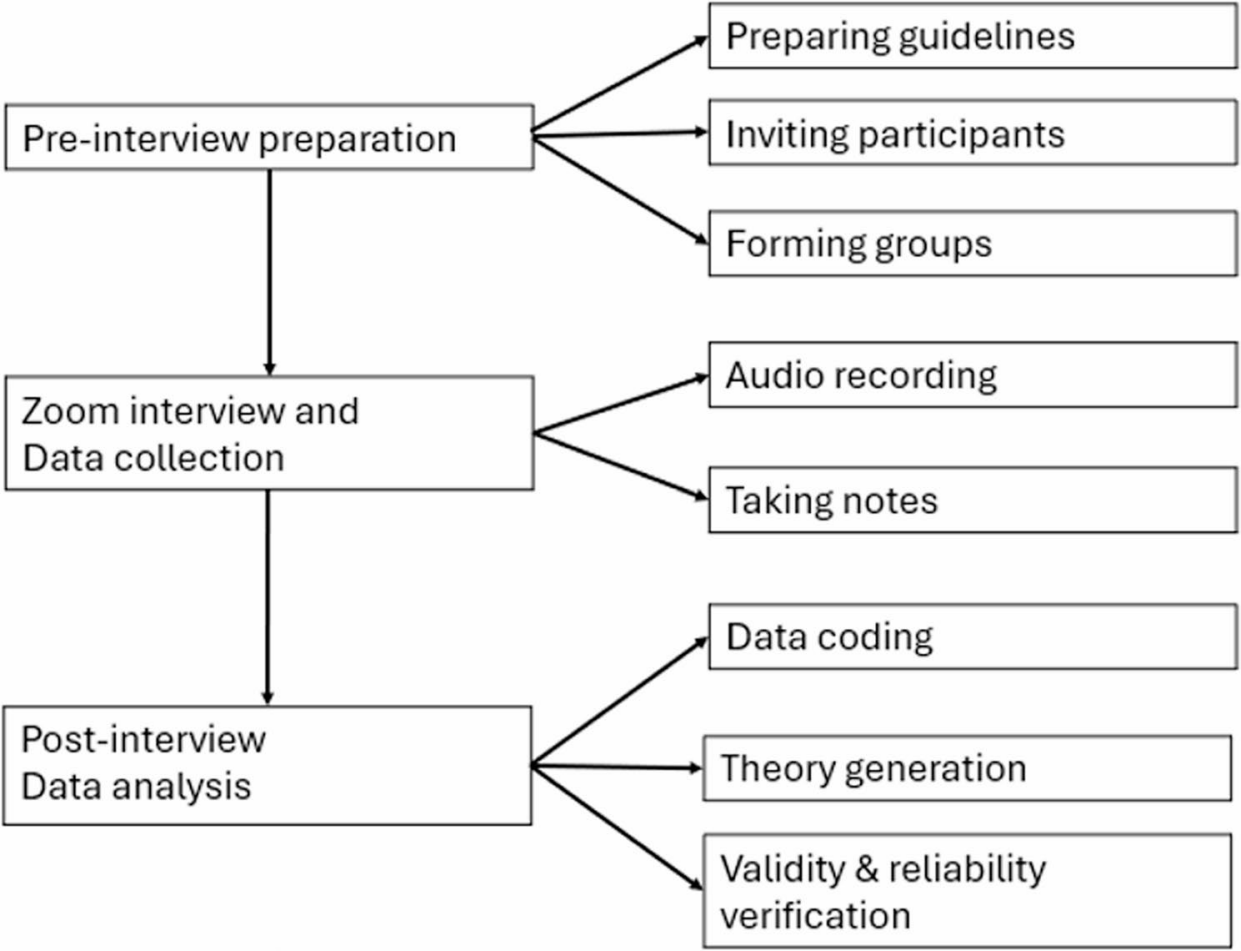
Flow-chart of focus group interview

The procedure of interview and data analysis is presented in Fig. 1. The semi-structured, focused-group interviews presented in this study took place 7–8 months after the end of the first intervention. Participants who finished the 6-month health coaching were invited for the interviews. Purposive sampling was used with regard to gender, age and lifestyle domains. Participation in the interview was voluntary. Interested participants were asked to provide their available time slots. Three focused-group interviews with a total of 18 participants (group A: 5 interviewees, group B: 7 interviewees, group C: 6 interviewees) were conducted by trained research assistants. Each group was interviewed for about 1 h via zoom. Two well-trained research assistants (GQ and LL) who hold a health-related master’s degree and had 5- to 10-year health-related research experience conducted the interviews. They piloted the interview questions and procedure in five middle-aged adults before the formal interviews and were supervised by the health psychologist (XY) who was experienced in qualitative studies. Prior to each interview, research assistants explained the purpose and procedure of the study and obtained informed consent from the interviewees. All interviews were audiotaped as most participants did not want to have their faces recorded. Field notes were made during and immediately after the interviews.

The semi-structured interviews followed an interview guide, which resulted in an iterative approach, for emerging themes and perspectives to be explored [29]. The semi-structured interviews were segmented into two fields of interest: (1) clients’ experience with lifestyle changes in general and during the intervention, (2) clients’ experience and views of health coaching (Table 1).

**Table 1:** Interview guide

Fields of interest	Probing questions
Experience with taking action for lifestyle change	Have you ever taken the initiative to change your lifestyle or continue with maintaining lifestyle changes? How did it go? What are the factors which have facilitated or hindered your lifestyle change or maintenance?
Experience and views of health coaching in lifestyle change	How did you feel about the health coaching? What have you found helpful or unhelpful in activating or maintaining your lifestyle change? How did you communicate with your health coach? How did you feel about the communication with your health coach?

After each interview, the audio recording was imported into Sonix AI for transcription. The research assistants then conducted a meticulous comparison verbatim between the AI-transcribed text and the audio recording to ensure the transcription accuracy and finalized the transcripts.

#### Coding and analysis

Inductive data analysis followed the six-stage thematic analysis approach by Braun and Clarke [30]. Transcripts were initially familiarized through repeated readings, segmented into data chunks, and labelled with codes. Codes were then organized into overarching themes, validated against the dataset, and defined with clear descriptions (Table 2). Finally, quotes exemplifying each theme were chosen for inclusion in the report using Microsoft Excel for data organization. The rigor of this study was ensured through careful measures to enhance credibility, transferability, dependability, confirmability, and reflexivity, based on recommendations in methodological papers [31]. To establish credibility, investigator triangulation was employed by involving two more researchers in coding, analysis, and interpretation decisions (implemented by XY and GQ, and reviewed by a third reviewer CW) (Table 2).

**Table 2:** Example of transcripts (Participant C1) taken during thematic analysis

Preliminary transcripts	Codes	Subtheme	Theme
“The first meeting should be face-to-face, as it allows coach and me to know each other. After we know each other, subsequent communication becomes easier, and we can utilize different forms of communication such as phone calls or WhatsApp, each with its advantages. People in Hong Kong, tend to be busy and prioritize work and family matters. Therefore, using WhatsApp is convenient as it allows for flexible communication.”	Phone calls or WhatsApp; Busy schedule; Flexibility	Comprehensive approach	Health coaching facilitators
“The health coach regularly checked whether I have been exercising and whether I have been following the dietary guidelines.”	Regularly check	Regular reminders (online & offline)	

## Results

### Background characteristics of the participants

Six men and twelve women, with an average age of 51.9 years old (ranging from 37 to 60 years old) were interviewed (Table 3). Seven of the participants focused on enhancing nutrition (healthy eating habits and food choices) as their primary target lifestyle domain during health coaching, seven participants on enhancing physical activity (improving and maintaining regular physical activity according to WHO’s recommendation), three participants on improving sleep quality (satisfaction with sleep experience), and one participant on improving personal relationships (low interpersonal conflict or loneliness and high interpersonal well-being).

**Table 3:** The background information of the interviewees (*n* = 18)

Participant	Age	Gender	Primary target lifestyle domain
A1	57	Male	Nutrition
A2	55	Female	Nutrition
A3	37	Male	Physical activity
A4	46	Female	Sleep quality
A5	59	Female	Sleep quality
B1	56	Female	Physical activity
B2	55	Female	Nutrition
B3	54	Female	Physical activity
B4	40	Female	Nutrition
B5	56	Female	Nutrition
B6	44	Female	Physical activity
B7	51	Male	Sleep quality
C1	60	Male	Nutrition
C2	50	Male	Physical activity
C3	56	Male	Physical activity
C4	58	Female	Personal relationships
C5	44	Female	Nutrition
C6	57	Female	Physical activity

### Social/family obligations

The interviewees mentioned various reasons as to why they had difficulties changing or maintaining healthy lifestyles. The most commonly mentioned barrier to regular exercises or healthy eating was social/family obligations (long working hours, communal meals). For example, A3 mentioned that “Due to my demanding workload, I often work overtime even during Saturdays. I have difficulties in consistently engaging in exercise or healthy diet.” “I have simple lunch, including cookies and drinks. Because I have so much work to do, I can’t spare an hour to sit in a restaurant and enjoy a meal.” (C6) She also mentioned communal meals as her barrier to maintain healthy eating and sleep: “During festivals and holidays like New Year, Mother’s Day, and friends’ birthdays, it is inevitable to have unhealthy diet where people dine together.” B2 viewed her role of housewife as a barrier: “If I pay great attention to the nutrition and minimizing oil and salt, other family members don’t like the taste. Their attitudes disappoint me.”

### Feelings of discomfort/visceral discomfort

Several interviewees mentioned feelings of discomfort/visceral discomfort to fight against previous habits. B4 mentioned “I have to avoid or reduce consumption of foods that I enjoy because they raise my cholesterol levels. If I want to improve my health condition, I have to change my dietary habits and eat less food I like. It is difficult at beginning because healthy food is often untasted.” B7 mentioned “I am lazy and don’t like physical activity. Even walking ten thousand steps a day makes me breathless.” “I have been trying to go to bed early. However, when I wake up at 2 a.m., it is distressing and difficult to fall back asleep.” (C6).

### Environment in Hong Kong

Two interviewees mentioned environment in Hong Kong as a barrier to maintaining regular physical activity or healthy eating. B3 considered weather in Hong Kong as a barrier: “It’s challenging for me to stick at exercise. Especially during winter, it rains or gets cold; my body can’t handle it.” “When it keeps raining, I have difficulties to maintain exercise habit.” (C3) A4 mentioned that the barbeque facilities in the neighborhood affects her eating habit: “I love barbeque. Since I live in a village house, barbequing is convenient.” She also mentioned the tradition of afternoon tea in Hong Kong: “I have a habit of having afternoon tea at least twice a week. I enjoy eating egg tarts, pineapple buns, or pineapple buns with butter.” While the local environment facilitated her high-salt, sugar, and oil eating habits, it appears that the feelings of comfort and joy from the food made her maintain such habits since she used the term “love” and “enjoy”.

### Self-regulation

The interviewees highlighted that self-regulation (behavioral regulation, emotional regulation) was the key to facilitating lifestyle change. A4 said that “My self-control is weak, and that’s one of the difficulties I face.” A3 mentioned that “Relying solely on self-control to consistently engage in exercise or pay attention to my diet is challenging. It requires strong willpower. Although other people can help, but the effect is relatively weak.”

### Small steps and nudges

Small steps and nudges were frequently mentioned as a facilitator to initiate and maintain the change. For example, A3 mentioned that “Previously, I hardly engaged in any exercise. However, after the health coaching project (We WATCH), now I started to run a little more and regularly which can help me to do a little bit more exercise.” B7 said “I did not have much time for exercise in daily life. During health coaching, I realized that exercise could be so simple, and I followed the plan including five-minute walk in the neighbourhood and mindfulness observation of the surrounding environment.” B1 also mentioned that “I started with brisk walking for about thirty minutes each day, and then gradually incorporated incline walking. In this way, I gradually increased the intensity of my exercise. Then I also started doing squats.” B4 said that “I have to gradually adjust my habits for the change. I reduce a little bit food I enjoy and consume a little bit more fruits and vegetables.”

### Personalised problem solving

Personalised problem solving was recognised as facilitator of taking action to change lifestyles. B2 said that “My health coach came up with solutions with me, such as using technology (e.g., remote control light) to solve my my sleep difficulty.” A3 mentioned that “My health coach suggested cherry tomatoes, which allows me eating more fruits/vegetables while working. It is smart and easy to follow.” Furthermore, such feasible and personalized goals were also important to facilitate long-term lifestyle change. As B1 mentioned, “Since joining the health coaching project (We WATCH), I can now walk briskly for thirty minutes every day. Due to my knee problem, running is not suitable for me; therefore, my health coach suggested brisk walking.”

### Review of body check results

Review of body check results helps to maintain motivation and accomplishment. For example, A4 mentioned that “The health indicators reminded me of the status of my body”. B2 said that “The body checks before and after 6-month coaching are beneficial as they allow me to check my body data and the effect of participating in the program.” “After first body check, I was so surprised that my triglyceride levels were significantly higher than the standard value, almost double. My total cholesterol level was also elevated. The post-program check showed my triglyceride levels significantly decreased to the normal level. I feel accomplished and successful in my progress.” (C2).

### Regular reminders

Regular reminders from health coaches were important cues to actions in lifestyle change. A4 mentioned that “My health coach used WhatsApp for my progress checking, whether I am able to continue, and whether I am ready to get rid of my unhealthy habits. When I indulged, the coach would remind me of aerobic exercises.” The continues monitor and reminders from health coaches helped to maintaining the new behaviour until the new behaviours became a habit. As A4 said, “The health coach consistently reminded me, which helped me to stay committed.” “After many reminders from the health coach, I genuinely started to make an effort to build up healthy dietary habits.” (B4) “The health coach regularly checked whether I have been exercising and whether I have been following the dietary guidelines.” (C1) Furthermore, the new habit forming is not only physical or behavioral but also in the mind. As B3 mentioned, “Throughout the process of health coaching, I have developed the habit of reminding myself to exercise and paying attention to my diet.”

### Lack of support after the project

However, two interviewees mentioned difficulties maintaining lifestyle changes without support when health coaching was not available after the project. “There are indeed challenges, and one of them is that there is no longer anyone monitoring me after the program.” (B6) “After the program, I feel directionless and unsure about how to further improve my physical condition. I’m not sure where to seek help or guidance.” (C2).

### E-health and digital device use

Most of the interviewees highlighted the importance of e-health and digital device use in reminding, enhancing their awareness, self-monitoring, and risk prevention. It may be promising solution for behaviour maintenance after the project. Thus, enhancing e-health is important in heath coaching in this digital age. “Using digital watch, I can check my previous records and progress for running. It can also motivate me to run more when I find I have insufficient exercise.” (A1) “I use the watch to track my heart rate, and found I have a risk of arrhythmia.” (B1) “The watch can play music, which is convenient during exercise…… I can run while enjoying the music. Running to the rhythm of the music makes me feel comfortable and easy to run more.” (B3) “The watch helped me to track my sleep quality.” (C6) However, two interviewees considered that digital watch was not helpful. “The data detected by the watch doesn’t hold much reference value. If you want to check your body indicators, it’s better to consult a doctor for a thorough body check.” (B1) “The record of my physical activity was not accurate.” (B2) Two interviewees mentioned that they were not good at using the watch. “I have trouble using this watch effectively.” (B2) “I’m not very familiar with using this watch.” (B5).

### Communication mode

Most interviewees preferred the combination of online and offline communication channels and found it helpful, while they recognized the advantages of disadvantages of each approach. “My coaching sessions at the beginning and end were in-person, while the intermediate sessions used WhatsApp, and I find this method suitable for me. This program aligns well with my preferences and needs.” (A4) “Communication in the form of phone calls might be better. Although face-to-face interaction is ideal, it may not always be possible to find the time for it. Phone calls can be a convenient option. A 5-minute phone conversation might be more ideal than just relying on WhatsApp for contact.” (A3) “The first meeting should be face-to-face, as it allows coach and me to know each other. After we know each other, subsequent communication becomes easier, and we can utilize different forms of communication such as phone calls or WhatsApp, each with its advantages. People in Hong Kong, tend to be busy and prioritize work and family matters. Therefore, using WhatsApp is convenient as it allows for flexible communication.” (C1).

### Supportive and trusting relationship

Supportive and trusting relationship with the health coach was often mentioned by the interviewees. The client-centred and supportive approach made the interviewees feel warm and cared for. “I feel that the health coach is really caring. They are polite and gentle. The advice they provide is very professional, and it makes me feel very supported. The health coach asks for my preferences and provides advice, accordingly, allowing us to establish a good rapport and making the entire process very comfortable.” (A4) “I have a great relationship with my health coach, and we have detailed communication.” (B2) “When I achieved the goals, they gave me affirmation. They are caring and set goals based on my personal needs and willingness.” (A2) “It’s incredibly caring and supportive.” (A5).

### Client-health professional relationships

**Fig. 2:**
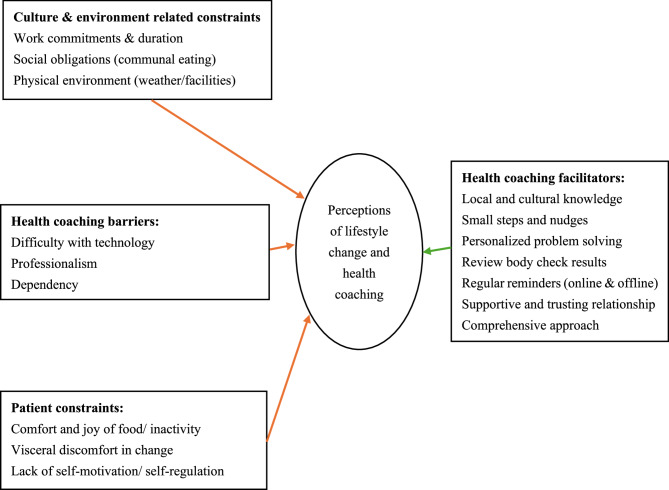
Experience of lifestyle change and health coaching in Hong Kong (barriers in left-side boxes and facilitators in right-side boxes)

Three interviewees highlighted that health coach is different from traditional healthcare service, including client-health professional relationships. “Doctors primarily focus on symptoms treatment. If I have high cholesterol, the doctor may prescribe medication to reduce it. Doctors may not always be able to solve the underlying problems. Health coaches pay attention to my overall well-being and lifestyles.” (B7) “Doctors are responsible for medical treatment, whereas health coaches work on disease prevention and overall well-being.” (A5) “My relationship with health coach is better than that with doctors. Doctors rarely spend ten minutes on a phone call with you.” (C3) One questioned health coaching professionalism. “I’m not sure if the advice given by the health coach is as professional as that of a traditional doctor.” (C5) (Fig. 2).

## Discussion

This is the first study exploring the barriers and facilitators of lifestyle behavioural change and maintenance after a health coaching program in a Chinese context (Fig. 2 for the summary generated from the findings). Findings of the present study suggest that adopting healthier lifestyle habits can be particularly challenging for middle-aged individuals in Hong Kong due to a combination of barriers. First, the demanding work culture in Hong Kong often leaves little time for exercise or meal preparation for middle-aged individuals who are often working populations. It can exacerbate the struggle to prioritize health amidst professional commitments. Second, the region’s humid and rainy climate presents environmental barriers to outdoor physical activities which can hinder opportunities for regular exercise and maintenance. Third, social obligations, deeply rooted in communal eating traditions, can make it difficult for individuals to resist unhealthy food choices during social gatherings or eating with families. These cultural/regional barriers may be also applied to other Asian or coastal regions with collectivism cultures (e.g., Singapore, Pakistan). For example, previous studies found that collectivism had a negative influence on diet-related behaviours in Punjabi Sikh men [32]. Negotiating changes in diet needs intertwined with family practices, gender divisions of labour, and collectivist ideals [32]. Thus, family-based health coaching that includes all family members for decision making and exploring indoor exercises during health coaching via techniques of problem solving and smart goal setting may be particularly helpful in such cultures.

Additionally, participants mentioned that the allure of familiar comfort foods (e.g., high-calorie food) and the inertia of inactivity can impede efforts to make or maintain behavioural changes. Overcoming these obstacles and making lifestyle changes are compounded by visceral discomfort. Changing lifestyles and establishing new habits require significant adjustments to daily routines and may arouse negative affective responses. This suggests a deep-rooted connection between lifestyles and emotions. The significant role of emotions in unhealthy lifestyles and lifestyle changes has been highlighted in previous surveys [33–35]. Thus, health coaching should adopt a multifaceted approach that considers not only the nutritional and physical aspects but also the psychological influences on behavioural change. Strategies could include personalized counselling or coaching to help individuals navigate emotional eating triggers, emotional regulations skills, motivational interventions to overcome inertia and initiate physical activity, and support systems that facilitate gradual lifestyle modifications to enhance long-term adherence.

One barrier of healthy lifestyle maintenance was related to the completion of the health coaching program when health coaching service is not available anymore. One solution is to cultivate clients’ autonomous self-regulation and self-efficacy that can have long-term effects on maintaining clients’ changes after projects [36, 37]. Health coaching should avoid making the clients over rely on their coaches for decision making. Instead, self-efficacy and empowering the clients to be responsible for their own lifestyles and wellness should the core of health coaching.

In promoting health lifestyle changes and maintenance, participants mentioned several facilitators related to health coaching to foster healthy behaviours. First, health coaches serve as guides and motivators, offering personalized support and expertise to facilitate sustainable lifestyle changes. Their regular progress checking not only provides accountability but also enables individuals to track their improvements and stay motivated on their health journey. Health coaching adopt the techniques of smart goal, personalized advice, and incorporating easy habits can help clients to initiate lifestyle changes. Enhancing clients’ self-awareness of their own health status through tools like blood testing and digital watches can empower clients to monitor their health metrics and make informed decisions about their well-being. Moreover, leveraging the digital watches beyond physical measurements to support emotional well-being—such as music playlists for relaxation, and tools for managing stress—can enhance overall mental and emotional health, complementing physical wellness efforts. Such digital devices may also help clients to maintain their healthy behaviours even after the health coaching program. Other lifestyle promotion programs including digital self-monitoring technologies also reported promising effects on changing and sustaining health-related behaviours [38].

Some characteristics of health coaches were highlighted by the clients that may affect the effect of health coaching. By demonstrating genuine care and compassion through their caring demeanour, holistic perspectives, and client-centred approach, health coaches create a supportive environment where clients feel heard and valued, fostering trust and openness in the coaching relationship [25]. In addition, such an approach considers the interconnected facets of clients’ lives—physical, mental, emotional, and social—recognizing that well-being encompasses more than just physical health and thus may facilitate clients’ behaviour change [39]. This may advocate a shift in the doctor-patient relationship from the ‘guidance-co-operation’ model to ‘mutual participation’, whereby power and responsibility are shared with the patient [40]. In Hong Kong, the relationship has been predominantly between a patient seeking help and a doctor whose decisions are silently complied with by the patient [41]. In this paternalistic model of the doctor-patient relationship, the doctor is in a position of power, where the patient is expected to cooperate and obey without question. Our health coaching program may promote the transition to the egalitarian model that emphasizes mutual participation between the doctor and patient, with equal power, mutual independence, and equal satisfaction. However, there were participants questioning the professionalism of health coaches or requesting direct guide from health professionals instead of shared decision making. It is reasonable as health coaching and health coaches are relatively new in Hong Kong, and our health coaching program represents the first attempt to establish evidence-based health coaching service for middle-aged adults [42]. There is no professional body for health coach certification locally like AFPA’s health coach certification in the United States [43]. Thus, efforts on health coaching promotion, local adaptation and acceptability enhancement in both clients and health professionals in Hong Kong are warranted. Such efforts should also aim to empower the clients to make their own decisions and be responsible for their health. Health coaching emphasizes partnership as coaches do not serve in the expert role with their clients. They are partners, and together, they explore the client’s world and how it can be improved. Thus, health coaches need to keep a balance in their expert role and partner role.

When considering modality used in health coaching in Hong Kong, most participants preferred a combination of online and offline channels which offers a versatile and comprehensive approach to reach and engage clients. Face-to-face meetings offer a personal touch and facilitate deeper connections between coach and clients which were preferred for the first meeting by the participants [44, 45]. On the other hand, online communication provides instant connectivity and wide reach, enabling real-time interactions and information dissemination. These digital channels are effective for sharing updates, announcements, and engaging with tech-savvy clients who prefer online interactions and have difficulty to find time for face-to-face coaching, especially for the middle-age working populations and Hong Kong culture where residents have a fast-paced lifestyle and long working hours. However, the efficacy of online versus face-to-face health coaching has been compared in previous studies and showed inconsistencies [44, 46, 47]. Local health coaching programs should take the clients’ preference and pragmatic issues into consideration and provide empirical data regarding the effectiveness of different communication modes.

There were limitations common to all qualitative studies. The specificity of verbal reports cannot be equated with quantitative outcomes, nor with mechanisms that explain associations between experience and outcomes (e.g., lifestyle changes). The context-specific nature of our qualitative findings may limit their applicability to other settings or populations. It is also challenging to validate or replicate our qualitative findings. Last but not least, while Zoom focused-group interviews offered convenience and flexibility for conducting research, researchers should be mindful of the limitations associated with this format, such as limited nonverbal cues and difficulty in managing group dynamics, establishing rapport, and maintaining interactions.

## Conclusions

These findings underscore the complex interplay between psychological, behavioural, and cultural/environmental factors that influence individuals’ health-related decision-making processes and the considerations for a multifaceted health coaching program. The findings can be used to guide the development of health coaching programs in Hong Kong and similar cultures. Health coaching in these cultures requires tailored strategies that acknowledge the unique cultural and environmental context (e.g., family-based health coaching, exploring indoor exercises).

## Supplementary Information

Supplementary Material 1.

Supplementary Material 2.

Supplementary Material 3.

Supplementary Material 4.

Supplementary Material 5.

## Data Availability

Data will be available by request. Please contact the corresponding author for the data.

## Abbreviations

WHO: World Health Organization
NGO: Non-governmental organization

## Declarations

### Clinical trial number

Chinese Clinical Trial Registry: ChiCTR2400092811 (Registration Date: November 25, 2024).
