# Supplementary figures and images for: Multifaceted health coaching intervention for cardiovascular risk prevention – exploratory qualitative study of Chinese clients' perspectives
Source: BMC Prim Care. 2025 Aug 4;26:242. doi: 10.1186/s12875-025-02957-0 (PMC12323232; doi:10.1186/s12875-025-02957-0)

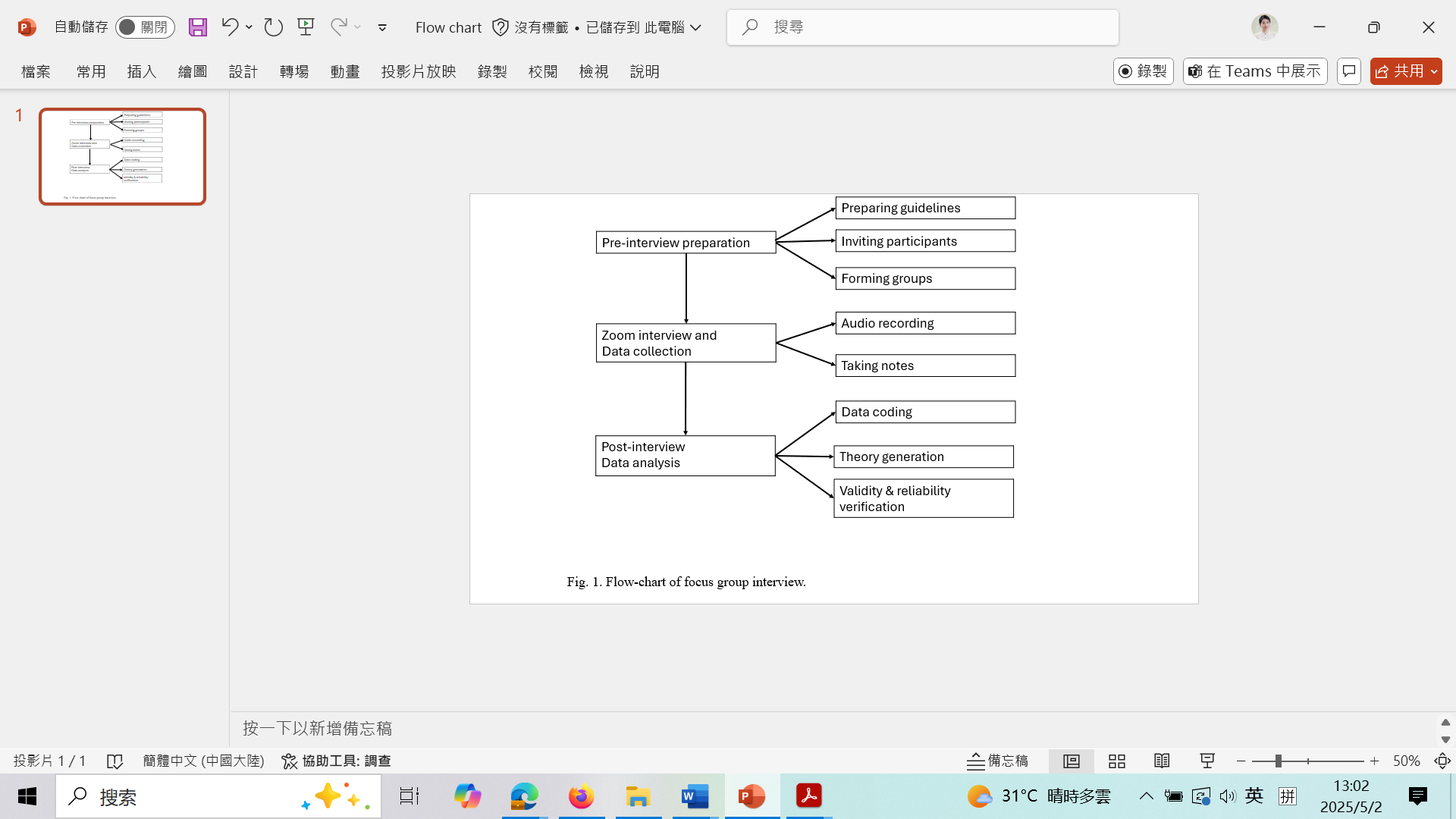


**Figure 1** Flow-chart of focus group interview

Supplement: Supplementary file 1 — Supplementary Material 1. [file 12875_2025_2957_MOESM1_ESM.docx]
