# Supplementary material for: Multifaceted health coaching intervention for cardiovascular risk prevention – exploratory qualitative study of Chinese clients' perspectives
Source: BMC Prim Care. 2025 Aug 4;26:242. doi: 10.1186/s12875-025-02957-0 (PMC12323232; doi:10.1186/s12875-025-02957-0)
Supplement: Supplementary file 2 — Supplementary Material 2. [file 12875_2025_2957_MOESM2_ESM.docx]

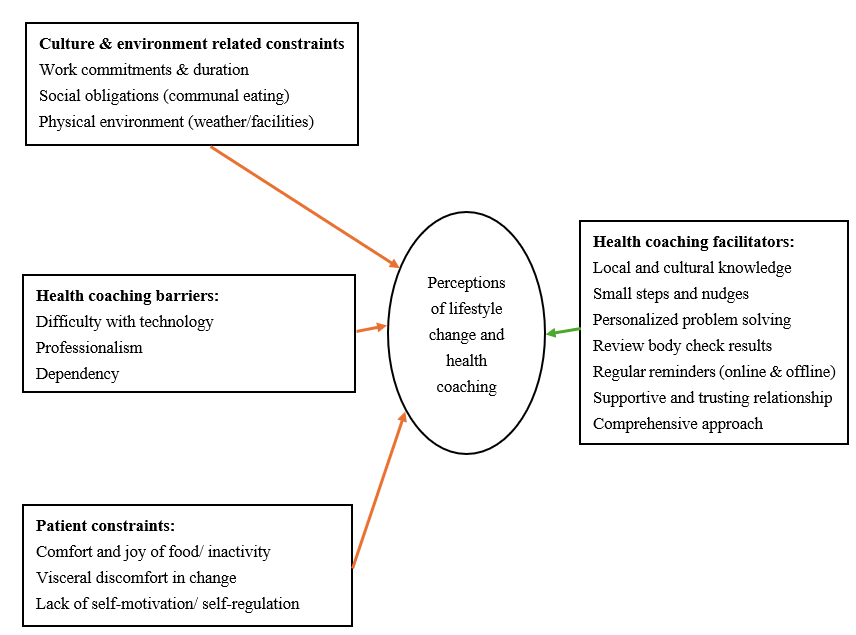


**Figure 2** Experience of lifestyle change and health coaching in Hong Kong (barriers in left-side boxes and facilitators in right-side boxes)
