# Supplementary material for: Multifaceted health coaching intervention for cardiovascular risk prevention – exploratory qualitative study of Chinese clients' perspectives
Source: BMC Prim Care. 2025 Aug 4;26:242. doi: 10.1186/s12875-025-02957-0 (PMC12323232; doi:10.1186/s12875-025-02957-0)
Supplement: Supplementary file 3 — Supplementary Material 3. [file 12875_2025_2957_MOESM3_ESM.docx]

**Table 1** Interview guide

| **Fields of interest** | **Probing questions** |
| --- | --- |
| Experience with taking action for lifestyle change | Have you ever taken the initiative to change your lifestyle or continue with maintaining lifestyle changes? How did it go? What are the factors which have facilitated or hindered your lifestyle change or maintenance? |
| Experience and views of health coaching in lifestyle change | How did you feel about the health coaching? What have you found helpful or unhelpful in activating or maintaining your lifestyle change? How did you communicate with your health coach? How did you feel about the communication with your health coach? |
