# Supplementary material for: Multifaceted health coaching intervention for cardiovascular risk prevention – exploratory qualitative study of Chinese clients' perspectives
Source: BMC Prim Care. 2025 Aug 4;26:242. doi: 10.1186/s12875-025-02957-0 (PMC12323232; doi:10.1186/s12875-025-02957-0)
Supplement: Supplementary file 4 — Supplementary Material 4. [file 12875_2025_2957_MOESM4_ESM.docx]

**Table 2** Example of transcripts (Participant C1) taken during thematic analysis

| **Preliminary transcripts** | **Codes** | **Subtheme** | **Theme** |
| --- | --- | --- | --- |
| “The first meeting should be face-to-face, as it allows coach and me to know each other. After we know each other, subsequent communication becomes easier, and we can utilize different forms of communication such as phone calls or WhatsApp, each with its advantages. People in Hong Kong, tend to be busy and prioritize work and family matters. Therefore, using WhatsApp is convenient as it allows for flexible communication.” | Phone calls or WhatsApp;  Busy schedule; Flexibility | Comprehensive approach | Health coaching facilitators |
| “The health coach regularly checked whether I have been exercising and whether I have been following the dietary guidelines.” | Regularly check | Regular reminders (online & offline) |  |
