# Supplementary material for: Multifaceted health coaching intervention for cardiovascular risk prevention – exploratory qualitative study of Chinese clients' perspectives
Source: BMC Prim Care. 2025 Aug 4;26:242. doi: 10.1186/s12875-025-02957-0 (PMC12323232; doi:10.1186/s12875-025-02957-0)
Supplement: Supplementary file 5 — Supplementary Material 5. [file 12875_2025_2957_MOESM5_ESM.docx]

**Table 2** The background information of the interviewees (n = 18)

| **Participant** | **Age** | **Gender** | **Primary target lifestyle domain** |
| --- | --- | --- | --- |
| A1 | 57 | Male | Nutrition |
| A2 | 55 | Female | Nutrition |
| A3 | 37 | Male | Physical activity |
| A4 | 46 | Female | Sleep quality |
| A5 | 59 | Female | Sleep quality |
| B1 | 56 | Female | Physical activity |
| B2 | 55 | Female | Nutrition |
| B3 | 54 | Female | Physical activity |
| B4 | 40 | Female | Nutrition |
| B5 | 56 | Female | Nutrition |
| B6 | 44 | Female | Physical activity |
| B7 | 51 | Male | Sleep quality |
| C1 | 60 | Male | Nutrition |
| C2 | 50 | Male | Physical activity |
| C3 | 56 | Male | Physical activity |
| C4 | 58 | Female | Personal relationships |
| C5 | 44 | Female | Nutrition |
| C6 | 57 | Female | Physical activity |
